# Investigation of SiO_2_ Etch Characteristics by C_6_F_6_/Ar/O_2_ Plasmas Generated Using Inductively Coupled Plasma and Capacitively Coupled Plasma

**DOI:** 10.3390/ma15041300

**Published:** 2022-02-10

**Authors:** Dain Sung, Long Wen, Hyunwoo Tak, Hyejoo Lee, Dongwoo Kim, Geunyoung Yeom

**Affiliations:** 1 Department of Advanced Materials Science and Engineering, Sungkyunkwan University, Suwon 16419, Korea; mintcheek@naver.com (D.S.); moon322223@naver.com (L.W.); stampede524@gmail.com (H.T.); hyejooyi@naver.com (H.L.); dwkim111@gmail.com (D.K.); 2 SKKU Advanced Institute of Nano Technology (SAINT), Sungkyunkwan University, Suwon 16419, Korea

**Keywords:** inductively coupled plasma (ICP), capacitively coupled plasma (CCP), liquid fluorocarbon (PFC), L-FC, C_6_F_6_, plasma etching, high aspect ratio contact (HARC)

## Abstract

The etching properties of C_6_F_6_/Ar/O_2_ in both an inductively coupled plasma (ICP) system and a capacitively coupled plasma (CCP) system were evaluated to investigate the effects of high C/F ratio of perfluorocarbon (PFC) gas on the etch characteristics of SiO_2_. When the SiO_2_ masked with ACL was etched with C_6_F_6_, for the CCP system, even though the etch selectivity was very high (20 ~ infinite), due to the heavy-ion bombardment possibly caused by the less dissociated high-mass ions from C_6_F_6_, tapered SiO_2_ etch profiles were observed. In the case of the ICP system, due to the higher dissociation of C_6_F_6_ and O_2_ compared to the CCP system, the etching of SiO_2_ required a much lower ratio of O_2_/C_6_F_6_ (~1.0) while showing a higher maximum SiO_2_ etch rate (~400 nm/min) and a lower etch selectivity (~6.5) compared with the CCP system. For the ICP etching, even though the etch selectivity was much lower than that by the CCP etching, due to less heavy-mass-ion bombardment in addition to an adequate fluorocarbon layer formation on the substrate caused by heavily dissociated species, highly anisotropic SiO_2_ etch profiles could be obtained at the optimized condition of the O_2_/C_6_F_6_ ratio (~1.0).

## 1. Introduction

As the semiconductor device size has decreased to nanoscale due to the high integration of the circuit, the critical dimension has decreased to a few nanometers and the device structure has changed from 2D to 3D. To fabricate these devices, etching technologies such as pulsed plasma etching technology and multiple patterning technology are widely investigated [1,2,3,4,5,6,7,8,9] and applied using two different plasma etching systems, namely the capacitively coupled plasma (CCP) etching system and inductively coupled plasma (ICP) etching system.

The CCP system uses two facing electrodes, and multiple RF powers composed of high and low RF frequencies are generally applied to both the top and bottom electrodes or to the bottom electrode only; to provide ion energy to the substrate located at the bottom electrode, a lower frequency RF power is generally connected to the bottom electrode. Due to the electric field configuration of the CCP system, the CCP system tends to show a low plasma density of ~109–10/cm^3^ and a low gas dissociation. In the case of the ICP system, one RF power is connected to the inductive antenna located at the top of the chamber for the generation of time-varying azimuthal electric fields in the process chamber for the high-density plasma formation of ~1010–11/cm^3^ with a significant gas dissociation, and a low-frequency RF power is also connected to the bottom electrode to provide ion bombardment to the substrate [10]. The ICP system is generally operated at a low pressure of a few millitorr, and etch rates are generally higher than those by the CCP system operated at a few tens of millitorr due to the higher ion flux and higher radical flux of the ICP system during the etching. However, for the high aspect ratio contact (HARC) etching of SiO_2_ using perfluorocarbon (PFC)-based etch gases, even though the etch rates are slower than the ICP system, the CCP system is generally used due to the higher etch selectivity over mask layers caused by low dissociation of fluorocarbon gases at the lower plasma density in addition to the higher ion bombardment energy at the same low-frequency power to the substrate.

Previously, to obtain higher etch selectivity over mask layers in addition to high HARC SiO_2_ etch rates, researchers have investigated using the ICP system instead of the CCP system for the HARC etching [11,12,13,14]. Li et al. used C_4_F_6_ gas for the etching of SiO_2_ masked with photoresist in an ICP system, and even though the etch selectivity of SiO_2_/PR was higher than conventional PFC gas of C_4_F_8_, the maximum etch selectivity was limited to 4 [11]. Nakamura et al. also etched SiO_2_ masked with photoresist in an ICP system with various PFC gases having different C/F ratios such as C_3_F_6_, C_4_F_6_, C_4_F_8_, and C_5_F_8_ and showed that the higher C/F ratio improved the etch selectivity but the maximum etch selectivity obtained with C_4_F_6_ was 2 [12]. Kim et al. used hydrofluoroether-based gases in the etching of SiO_2_ with an ICP system, but no etch selectivity data were provided [13]. Therefore, even though studies have been carried out to etch SiO_2_ selectively to mask layers using the ICP system, no sufficient investigation has been carried out and no results applicable to HARC SiO_2_ etching have been reported.

During the etching of SiO_2_ using the PFC gases, PFC gases are dissociated/ionized, and the characteristics of the dissociation/ionization affect the etch characteristics of SiO_2_ such as etch rates, etch selectivity over mask layers, and etch profile. Because the characteristics of the dissociation/ionization of the PFCs by ICP and CCP systems are different, the HARC SiO_2_ etch characteristics will be different; however, currently, no detailed research results can be found in the literature on the differences between the ICP system and the CCP system for the dissociation/ionization of PFCs and their relationship to the etching of HARC SiO_2_ using the same PFC gases. Moreover, the reason for the use of the CCP system for the HARC etching instead of the ICP system is due to the lower dissociation of the fluorocarbon gas to have high C/F ratio radicals/ions in the plasma for the higher etch selectivity over mask layers, but no research has been carried out using a perfluorocarbon gas with the high C/F ratio ≥ 1.0 such as C_6_F_6_ for higher C/F ratio radicals/ions even for the higher gas dissociation. In this study, using a PFC gas having a high C/F ratio of C_6_F_6_, the differences in the plasma characteristics of the C_6_F_6_ and the etch characteristics such as etch rate, etch selectivity over a hard mask layer, and etch profile were compared between the ICP system and the CCP system. In addition, the possibility of using the ICP system in HARC SiO_2_ etching masked with an amorphous carbon layer (ACL) was investigated. In the case of C_6_F_6_, in addition to a high C/F ratio, the global warming potential (GWP) is very low compared to some of PFC gases generally used for HARC SiO_2_ etching such as CF_4_ and C_4_F_8_ [15,16,17], as shown in Table 1, and its boiling point is lower than 0 °C (a liquid PFC); therefore, if required, the undissociated C_6_F_6_ can be collected at the exhaust line, which can reduce the global warming effect further.

## 2. Materials and Methods

**Figure 1:**
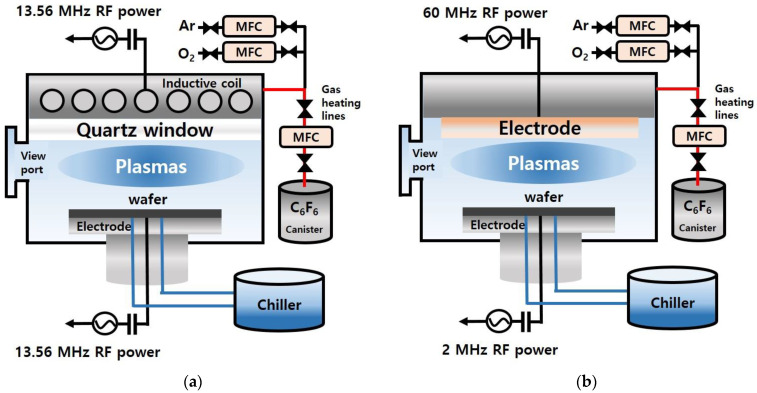
Schematic drawings of (**a**) the inductively coupled plasma (ICP) etching system and (**b**) the capacitively coupled plasma (CCP) etching system used in this experiment with C_6_F_6_ (liquid state at RT)/Ar/O_2_.

Figure 1 shows the schematics of the ICP etching system and CCP etching system used in the experiment. The ICP system used in this study was composed of a spiral gold-coated copper coil separated by a 1 cm thick quartz window located on the top of the process chamber for the generation of ICP with a 13.56 MHz RF power and the substrate holder biased with a separate 13.56 MHz RF power for the control of the ion energy to the substrate. The distance between the ICP source and the substrate was ~7 cm, and the ICP antenna and substrate were also cooled at room temperature by a chiller. In the case of the CCP system, one 60 MHz RF power was connected to the top electrode with a showerhead for the control of plasma density, and a 2 MHz RF power source was connected to the bottom electrode holding the substrate for the control of ion energy to the substrate. The distance between the two electrodes was ~30 mm, and both top and bottom electrodes were cooled at room temperature by a chiller. For the vacuum environments, a turbo molecular pump backed by a dry pump was used for both systems. To use liquid state C_6_F_6_, heating systems were connected to the gas lines of ICP and CCP systems. C_6_F_6_ (boiling point: 76 °C) was vaporized and delivered from a canister to the process chambers. Gas lines were maintained at 80 °C using heating systems. A quartz viewport window was located on the sidewall of the process chambers for the plasma analysis using an optical emission spectrometer (OES).

As the sample, 2.4 μm thick SiO_2_ on Si masked by 1.4 μm thick ACL with 160 nm hole patterns was used. In addition to C_6_F_6_, Ar and O_2_ were added for the processing, and the O_2_ flow rates were varied to control the fluorocarbon layer formed on the sample surface. For the ICP system, 500 W of RF power and −1500 V of bias voltage were applied to the ICP source and substrate while keeping 6 mTorr of operating pressure, and oxygen flow rate was varied from 15 to 25 sccm while keeping the gas flow rates of C_6_F_6_ and Ar at 20 and 10 sccm. In the case of the CCP system, 400 W of RF power and −1700 V of bias voltage were applied to the top electrode and substrate while keeping 30 mTorr of operating pressure, and the oxygen flow rate was varied from 150 to 200 sccm while maintaining the gas flow rates of C_6_F_6_ and Ar at 50 and 150 sccm.

The etch depths of SiO_2_/ACL and the SiO_2_ etch profiles were observed using field emission scanning electron microscopy (FE-SEM, Hitachi S-4700) after the etching using ICP and CCP, and the etch rates and etch selectivity were calculated. The compositions and binding states of the etched SiO_2_ surfaces were observed using X-ray photoelectron spectroscopy (XPS, Fisons Instruments Surface Systems ESCALAB 220i). Then, to fit the spectrum of the C1s peak and to calculate the atomic percentages on the SiO_2_ surfaces, an XPS peak-fitting software (software name: Thermo Scientific Avantage) and the Origin program (Origin Lab Corporation) were used. The differences in the dissociation species of C_6_F_6_ in the ICP and CCP used in our experimental conditions were observed using an optical emission spectroscope (OES, Isoplane SCT3200, Andor iStar 734).

## 3. Results and Discussion

**Figure 2:**
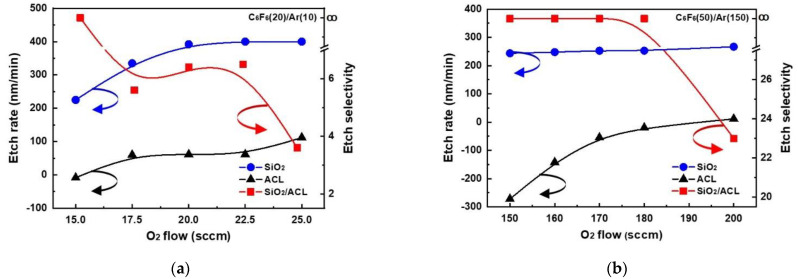
SiO_2_ etch rates and SiO_2_ etch selectivities over ACL in C_6_F_6_/Ar/O_2_ plasmas as a function of O_2_ gas flow rate in (**a**) ICP and (**b**) CCP systems. The flow rates of C_6_F_6_ and Ar were 20 and 10 sccm for the ICP system and 50 and 150 sccm for the CCP system.

Using C_6_F_6_/Ar/O_2_, ACL-masked SiO_2_ was etched in the ICP and CCP systems, and the etch rates and etch selectivities estimated from the etch depths of ACL and SiO_2_ are shown in Figure 2a for the ICP system and Figure 2b for the CCP system. As shown in Figure 2a, for the ICP system, until the oxygen flow rate was higher than 15.0 sccm, no etching of ACL was observed, and from the oxygen flow rate higher than 17.5 sccm, the ACL etch rate was increased with oxygen flow rate. In the case of SiO_2_, the etching was observed even at 15.0 sccm, and with an increase in the oxygen flow rate, the SiO_2_ etch rate was increased to ~400 nm/min at 20 sccm, and the further increase in oxygen flow rate nearly saturated the SiO_2_ etch rate. The etch selectivity of SiO_2_/ACL was infinite at 15 sccm and ~6.5 at 20–22.5 sccm, and the further increase in oxygen flow rate to 25 sccm decreased the etch selectivity to ~3.6. In the case of the CCP system, for the oxygen flow rate from 150 to 180 sccm, deposition of a fluorocarbon layer instead of etching was observed on the ACL, and when the oxygen flow rate was 200 sccm, ~12 nm/min of ACL etching was observed. In the case of SiO_2_, the etch rate ~244 nm/min was observed even at 150 sccm; with increasing oxygen flow rates, the etch rate was slightly increased (or nearly saturated) to 200 sccm by showing the etch rate of ~267 nm/min. However, when the oxygen flow rate was lower than 150 sccm, SiO_2_ etching was also decreased to ~0 for the CCP system (not shown). Therefore, the etch selectivity of SiO_2_/ACL was infinite from 150 to 180 sccm, and the etch selectivity of ~23 could be obtained at 200 sccm. From the above results, it can be concluded that the etching of SiO_2_ requires a much lower ratio of O_2_/C_6_F_6_ (0.75–1.25 for ICP and 3–4 for CCP) for the ICP system, the maximum SiO_2_ etch rate is higher for the ICP system (~400 nm/min for ICP and ~267 nm/min for CCP), and the etch selectivity is much higher for the CCP system at the maximum SiO_2_ etch rate (~6.5 for ICP and ~23 for CCP).

**Figure 3:**
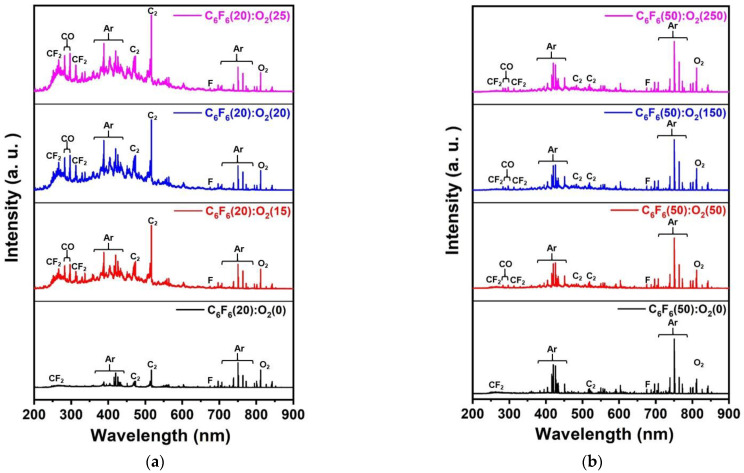
OES spectra of C_6_F_6_/Ar/O_2_ plasmas: (**a**) C_6_F_6_(20)/Ar(10)/O_2_(0–25) sccm for the ICP system and (**b**) C_6_F_6_(50)/Ar(150)/O_2_(0–250) sccm for the CCP system. The spectral peaks were normalized by the Ar peak intensity (750.4 nm).

**Figure 4:**
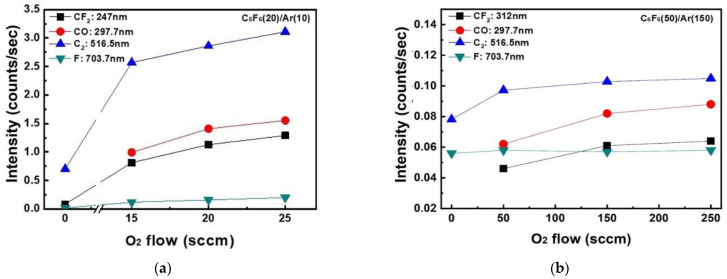
OES intensities of CF_2_ (247–312 nm), CO (297.7 nm), C_2_ (516.5 nm), and F (703.7 nm) normalized by Ar (750.4 nm) as a function of O_2_ gas flow rate for (**a**) the ICP system and (**b**) the CCP system.

To understand the differences in the etch characteristics of ACL-masked SiO_2_ with C_6_F_6_/Ar/O_2_ gas mixtures between the ICP and CCP systems, the dissociation characteristics of C_6_F_6_/Ar/O_2_ for different oxygen flow rates were investigated with OES for the wavelength range of 200–900 nm, and the results are shown in Figure 3a for the ICP system from 0 to 25 sccm of oxygen flow rate and Figure 3b for the CCP system from 0 to 250 sccm of oxygen flow rate. For easier comparison, the OES data were normalized by the Ar peak at 750.4 nm. As shown in Figure 3a,b, optical emission peaks from O (777.3, 844.8 nm), F (685.7, 703.8 nm), Ar (696,7, 706.8, 738.6, 750.4, 772.5, 794.9, 800.7, 801.6, 811.6, and 826.5 nm), CO (282.7, 296.8 nm), C_2_ (473.7, 516.7, 563.6 nm), CF_2_ (245–321 nm), etc., could be observed [19]. Among these peaks, the emission peaks related to CF_2_ (247 nm), CO (297.7 nm), C_2_ (516.5 nm), and F (703.7 nm) normalized by Ar (750. 4 nm) measured as a function of oxygen flow rate are shown in Figure 4a for the ICP system and b for the CCP system. In general, during the etching of SiO_2_ by fluorocarbon plasmas, F radicals react with Si on the SiO_2_ surface and are removed from the surface as SiF_x_ while C radicals react with O on the SiO_2_ surface and are removed from the surface as CO_x_ [20]. As shown in Figure 4a,b, the increase in densities of radicals such as CF_2_, CO, C_2_, and F with increasing oxygen flow rate could be observed in both the ICP system and the CCP system due to the increased dissociation of fluorocarbon by oxygen radicals in the plasmas. However, a more significant increase in the radicals with increasing oxygen flow rate was observed for the ICP system even with lower oxygen flow rates. When the radical ratios of CF_2_/F, CO/F, and C_2_/F were compared between the ICP and CCP systems, significantly high ratios of CF_2_/F (5.0–7.1 for ICP and 0.8–1.1 for CCP), CO/F (0–8.8 for ICP and 0–1.5 for CCP), and C_2_/F (15.5–41.5 for ICP and 1.4–1.8 for CCP) were observed for the ICP system due to the significantly higher radical densities of CF_2_, CO, and C_2_ compared to F density for the ICP system, indicating more significant dissociation of the C_6_F_6_ of the ICP system compared to the CCP system.

**Figure 5:**
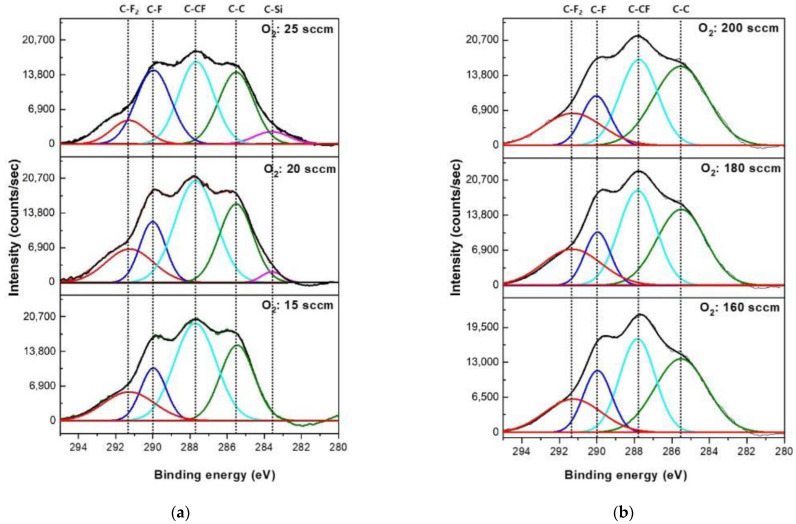
C1s XPS narrow scan spectra of SiO_2_ surface after etching using C_6_F_6_/O_2_/Ar plasmas as a function of O_2_ gas flow rates with (**a**) the ICP system and (**b**) the CCP system.

Figure 5 shows the XPS narrow scan data of C 1s on the SiO_2_ surfaces etched with C_6_F_6_/Ar/O_2_ for different oxygen flow rates for (a) the ICP system with C_6_F_6_(20)/Ar(10)/O_2_ (15–25 sccm) and (b) the CCP system with C_6_F_6_(50)/Ar(150)/O_2_(160~200 sccm). The other conditions are the same as those in Figure 2. The carbon bonding peaks such as C–C (285.5 eV), C–CF (287.8 eV), C–F (290.0 eV), and C–F_2_ (291.3 eV) could be observed [21]. For the ICP system, as shown in Figure 5a, the increase in oxygen flow rate from 15 to 25 sccm decreased C–C bonding and C–CF bonding slightly possibly due to the greater dissociation of species from the plasmas as shown in Figure 4a. In the case of the CCP system, the increase in oxygen flow rate from 160 to 200 sccm did not change the carbon bonding peaks on the SiO_2_ surface noticeably possibly due to no significant change of radical ratios with increasing oxygen flow rate as shown in Figure 4b. When C 1s bonding peaks on the etched SiO_2_ surfaces between the ICP system and the CCP systems are compared, the SiO_2_ surfaces etched by the CCP system show slightly higher C-CF bonding intensities compared to those by the ICP system. Table 2 shows the composition of the SiO_2_ surface etched using C_6_F_6_/Ar/O_2_ as a function of O_2_ gas flow rates for the ICP and CCP systems for the conditions in Figure 5. As shown in Table 2, even though the ratio of C_6_F_6_/O_2_ in the gas mixture was much higher for the SiO_2_ etching using the CCP system, similar compositions of fluorocarbon polymer layers were observed between the ICP system and the CCP system on the etched SiO_2_ surfaces; however, a more significant increase in Si percentage (0% to 2.5% for ICP and 0% to 0.7% for CCP) and a more significant decrease in C percentage (48.6% to 42.6% for ICP and 47.8% to 45.7% for CCP) were observed on the etched SiO_2_ surfaces for the ICP system compared to those for the CCP system, indicating thinner fluorocarbon layers on the SiO_2_ surface etched by the ICP system, which could increase SiO_2_ etch rate with increasing oxygen flow rate. In fact, the SiO_2_ etch rate was increased with oxygen flow rate from 15 to 22.5 sccm for the ICP system, while that for the CCP system was almost saturated with increasing oxygen flow rate from 150 to 200 sccm.

**Figure 6:**
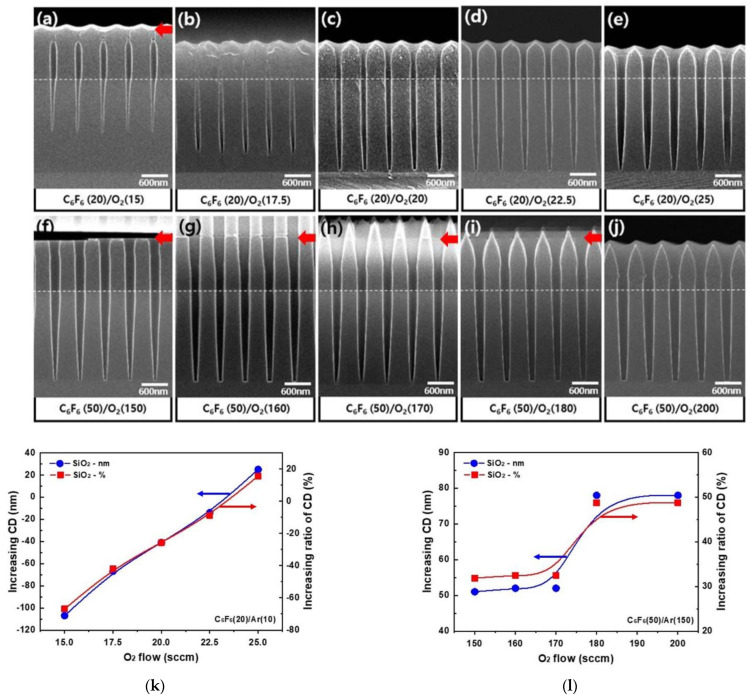
Etch profiles of 160 nm ACL hole masked SiO_2_ with different O_2_ gas flow rates of C_6_F_6_(20)/Ar(10)/O_2_(15–25 sccm) for the ICP system (**a**–**e**) and with C_6_F_6_(50)/Ar(150)/O_2_(150–200 sccm) for the CCP system (**f**–**j**). (**k**,**l**) The changes of pattern hole width (CD) and its changed percentages of SiO_2_ after the etching of ~2.4 μm thick SiO_2_ on Si masked by 1.4 μm thick ACL with 160 nm hole patterns for (**a**–**e**) the ICP system and (**f**–**j**) the CCP system, respectively, except for the cases when the ACL mask holes were clogged by a fluorocarbon layer. White dotted lines are the ACL mask bottom positions and red arrows show ACL mask top positions.

The etch profiles of 160 nm ACL hole masked SiO_2_ with different O_2_ gas flow rates of C_6_F_6_(20)/Ar(10)/O_2_(15–25 sccm) for the ICP system are shown in Figure 6a–e, and those with C_6_F_6_(50)/Ar(150)/O_2_(150–200 sccm) for the CCP system are shown in Figure 6f–j. White dotted lines are the ACL mask bottom positions and red arrows show ACL mask top positions. For the ICP etching with C_6_F_6_/Ar/O_2_, for 15–17.5 sccm of oxygen flow rate, the ACL mask was clogged by the fluorocarbon layer after etching 1.4–2.0 μm depth of SiO_2_. After using the oxygen flow rate of 20 sccm or higher, ~2.4 μm thick SiO_2_ could be fully etched with highly anisotropic etch profiles even though the etched amount of ACL mask was increased with the increase in oxygen flow rate. In the case of the CCP etching with C_6_F_6_/Ar/O_2_, for the oxygen flow rates of 150–180 sccm, a thick fluorocarbon layer was deposited on the top of the ACL mask while etching ~2.4 μm thick SiO_2_ anisotropically; therefore, infinite etch selectivity of SiO_2_/ACL could be obtained. For the oxygen flow rate of 200 sccm, the top of the ACL mask was etched while etching ~2.4 μm thick SiO_2_. Therefore, as shown in Figure 2b, the infinite etch selectivity of SiO_2_/ACL was observed for the oxygen flow rate of 150–180 sccm, and the etch selectivity of 23 was observed for the oxygen flow rate of 200 sccm. However, for the SiO_2_ etching with the CCP system, even though deposition of a fluorocarbon layer on the top of the ACL was observed during the etching of SiO_2_, the ACL sidewall was etched for the oxygen flow rates of 150–180 sccm; therefore, triangular-shaped ACL was observed after the etching.

The changes of SiO_2_ hole diameter critical dimension (CD) and its changed percentages after the etching of ~2.4 μm thick SiO_2_ on Si masked by 1.4 μm thick ACL with 160 nm hole patterns for Figure 6a–e the ICP system and Figure 6f–j the CCP system were showed in Figure 6k,l, respectively, except for the cases when the ACL mask holes were clogged by a fluorocarbon layer. As shown in Figure 6k, for the ICP system, due to the clogging of the ACL mask top area at the lower oxygen flow rates, the hole pattern diameter smaller than the ACL mask hole diameter was observed for the oxygen flow rate from 15 to 20 sccm (from −66% at 15 sccm to −25% at 20 sccm), but due to the increase in hole diameter with increasing oxygen flow rate, the hole diameter equal to the ACL mask hole diameter CD or higher was observed with the further increase in oxygen flow rate from 22.5 to 25 sccm (from −8% at 22.5 sccm to +15% at 25 sccm). Therefore, for the oxygen flow rate of 20~22.5 sccm, highly anisotropic SiO_2_ etch profiles with no significant hole diameter CD variation were observed for the etching by the ICP system with C_6_F_6_/Ar/O_2_, which could be applied for the HARC SiO_2_ etching. However, as shown in Figure 6l, for the etching by the CCP system with C_6_F_6_/Ar/O_2_, even though the etch selectivity of SiO_2_/ACL was infinite, due to the etching of ACL sidewall, for the oxygen flow rates of 150~200 sccm, an increase in hole diameter percentage from +31% to +48% was observed with less anisotropic SiO_2_ etch profiles compared to those etched by the ICP system.

**Figure 7:**
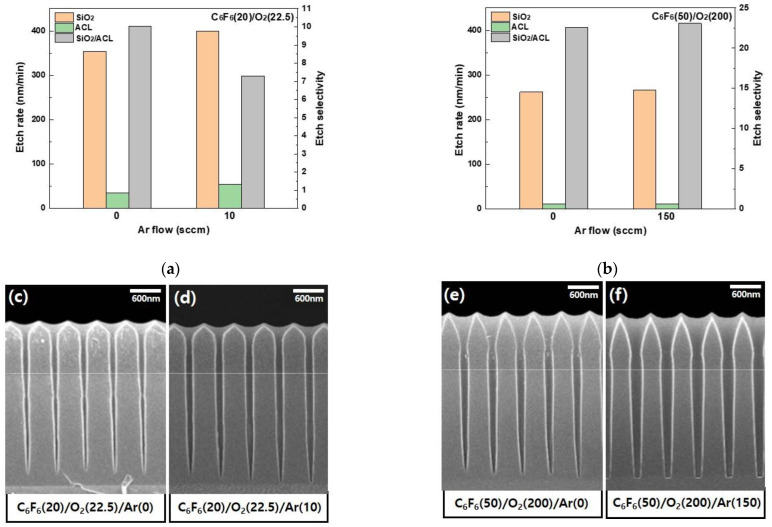
Etch rates of SiO_2_ and ACL and the etch selectivity of SiO_2_/ACL for (**a**) the ICP system and (**b**) the CCP system with and without Ar flow in C_6_F_6_/Ar/O_2_. (**c**,**d**) The SEM etch profiles of ACL-masked SiO_2_ with and without Ar flow for the ICP etching, respectively; (**e**,**f**) those with and without Ar flow for the CCP etching, respectively. The gas mixtures of C_6_F_6_(20)/Ar(0)/O_2_(22.5) and C_6_F_6_(20)/Ar(10)/O_2_(22.5) were used for the ICP etching and the gas mixtures of C_6_F_6_(50)/Ar(0)/O_2_(200) and C_6_F_6_(50)/Ar(150)/O_2_(200) were used for the CCP etching. White dotted lines in (**c**–**f**) are ACL mask lines and red dotted circle shows the bowed area in the SiO_2_ hole.

For the CCP system, the ratio of Ar/C_6_F_6_ used in the etching of ACL-masked SiO_2_ was much higher than that for the ICP system (10/20 for ICP and 150/50 for CCP); therefore, the widening of the SiO_2_ hole diameter CD could be related to the enhanced ion bombardment by Ar^+^ ions for the CCP system. In fact, in the experiment, the different Ar flow rates used for the ICP and CCP systems were related to the optimized process for the SiO_2_ etching with the ICP and CCP systems. However, to clearly show the effect of Ar gas flow rate on the etching, the SiO_2_ masked by ACL mask was also etched with and without the Ar flow rates for each of the ICP/CCP etching conditions. Figure 7 shows the etch rates of SiO_2_ and ACL and the etch selectivity of SiO_2_/ACL for (a) the ICP system and (b) the CCP system with and without Ar flow in C_6_F_6_/Ar/O_2_. (c) and (d) are the SEM etch profiles of ACL-masked SiO_2_ with and without Ar flow for the ICP etching, respectively, and (e) and (f) are those with and without Ar flow for the CCP etching, respectively. The gas mixtures of C_6_F_6_(20)/Ar(0)/O_2_(22.5) and C_6_F_6_(20)/Ar(10)/O_2_(22.5) were used for the ICP etching, and the gas mixtures of C_6_F_6_(50)/Ar(0)/O2(200) and C_6_F_6_(50)/Ar(150)/O2(200) were used for the CCP etching. As shown in Figure 7, the etch rates of SiO_2_ and the etch selectivities of SiO_2_/ACL were not significantly different for the etching with and without Ar flow for both the ICP system and the CCP system. In fact, there were some differences in the SiO_2_ etch profiles between the gas mixtures with and without Ar, as shown in Figure 7c–f; however, also, no significant differences could be observed. Therefore, the more significant etching of the ACL sidewall and the more significant widening of SiO_2_ hole diameter for the CCP system with C_6_F_6_/Ar/O_2_ are not related to the higher Ar+ ion bombardment flux to the substrate due to the higher Ar gas flow rate in the gas mixture. In fact, in the case of the CCP etching, due to the low dissociation of C_6_F_6_, higher-mass ions such as C_x_F_y_+ (x = 3–6, y = 4–5) having the ion mass higher than 100 (while the mass of Ar+ is ~40) can exist in the plasmas more than those formed during the ICP etching. The ion bombardment by those partially dissociated high-mass ions can etch the ACL sidewall more and widen the SiO_2_ hole diameter more, which can result in more tapered etch profiles as shown in Figure 6f–j.

## 4. Conclusions

In this study, using a perfluorocarbon (PFC) gas having a high C/F ratio of C_6_F_6_ and low GWP of 7, the differences in the plasma characteristics and the etch characteristics were compared between an ICP system and a CCP system. In addition, the possibility of using an ICP system instead of a conventional CCP system in HARC SiO_2_ etching masked with ACL was investigated for C_6_F_6_/Ar/O_2_ gas mixtures. It was found that, due to the higher dissociation of C_6_F_6_ and O_2_ for the ICP system compared to the CCP system, the SiO_2_ etching required a much lower ratio of O_2_/C_6_F_6_ while showing a higher maximum SiO_2_ etch rate and lower etch selectivity. However, in the case of the ICP etching with C_6_F_6_, even though the etch selectivity was much lower than that of the CCP etching with C_6_F_6_, due to less heavy-mass-ion bombardment in addition to an adequate fluorocarbon layer formation on the substrate, highly anisotropic SiO_2_ etch profiles could be obtained at certain oxygen gas flow rates added to C_6_F_6_. The high gas dissociation of the ICP system could be identified from higher densities of CF_2_, CO, and C_2_ radicals in the ICP system compared to the CCP system. It is believed that the ICP system could be also applied to the HARC SiO_2_ etching with higher etch rates and vertical etch profiles by using high C/F ratio PFCs and by controlling etch parameters adequately.

## Figures and Tables

**Table 1:** The lifetimes and 100-year GWPs of perfluorocarbon gases CF_4_, c-C_4_F_8_, C_2_F_6_, and C_6_F_6_ based on the IPCC reports [18].

Gas Species	Chemical Formula	Lifetime (Years)	GWP_100 years_
Perfluoromethane	CF_4_	50,000	6500
Perfluorocyclobutane	c-C_4_F_8_	3200	8700
Perfluoroethane	C_2_F_6_	10,000	9200
Hexafluorobenzene	C_6_F_6_	0.23	7

**Table 2:** Composition of the SiO_2_ surface etched using C_6_F_6_/Ar/O_2_ as a function of O_2_ gas flow rate for the ICP and CCP systems.

System Type	Etch Gas	Si 2p (%)	O 1s (%)	F 1s (%)	C 1s (%)	Total (%)
ICP	C_6_F_6_(20)/O_2_(15)	-	4.6	46.8	48.6	100
	C_6_F_6_(20)/O_2_(20)	1.1	5.5	45.8	47.6	100
	C_6_F_6_(20)/O_2_(25)	2.5	7.1	47.7	42.6	100
CCP	C_6_F_6_(50)/O_2_(160)	-	6.9	45.3	47.8	100
	C_6_F_6_(50)/O_2_(180)	-	7.0	46.0	47.0	100
	C_6_F_6_(50)/O_2_(200)	0.7	7.4	46.2	45.7	100

## Data Availability

All the data is available within the manuscript.

## References

[1] Kwon B., Kim J., Lee N., Shon J. (2010). Ultrahigh selective etching of SiO_2_ using an amorphous carbon mask in dual-frequency capacitively coupled C_4_F_8_/CH_2_F_2_/O_2_/Ar plasmas. J. Electrochem. Soc..

[2] Son J., Efremov A., Chun I., Yeom G., Kwon K. (2014). On the LPCVD-formed SiO_2_ Etching mechanism in CF_4_/Ar/O_2_ inductively coupled plasmas: Effects of gas mixing ratios and gas pressure. Plasma Chem. Plasma Process..

[3] Lee H., Yang K., Kim S., Shin Y., Suh D., Song H., Lee N., Yeom G. (2018). SiO_2_ etch characteristics and environmental impact of Ar/C_3_F_6_O chemistry. J. Vac. Sci. Technol. A.

[4] Samukawa S., Mukai T. (2000). High-performance silicon dioxide etching for less than 0.1-mm-high-aspect contact holes. J. Vac. Sci. Technol. B.

[5] Huang S., Huard C., Shim S., Nam S., Song I., Lu S., Kushner M. (2019). Plasma etching of high aspect ratio features in SiO_2_ using Ar/C_4_F_8_/O_2_ mixtures: A computational investigation. J. Vac. Sci. Technol. A.

[6] Cha T., Kim Y., Lee S., Cho Y., Chae H. (2019). Low-global warming potential fluoroether compounds for plasma etching of SiO_2_ and Si_3_N_4_ layers. J. Vac. Sci. Technol. A.

[7] Cho C., You K., Kim S., Lee Y., Lee J., You S. (2021). Characterization of SiO_2_ etching profiles in pulse-modulated capacitively coupled plasmas. Materials.

[8] Huang S., Shim S., Nam S.K., Kushner M. (2020). Pattern dependent profile distortion during plasma etching of high aspect ratio features in SiO_2_. J. Vac. Sci. Technol..

[9] Samukawa S. (2000). High-performance and damage-free plasma etching processes for future ULSI patterning. Microelectron. Eng..

[10] Nojiri K. (2015). Handbook of Dry Etching Technology for Semiconductors.

[11] Li X., Hua X., Oehrlein G. (2002). Fluorocarbon-based plasma etching of comparison of and discharges. J. Vac. Sci. Technol. A.

[12] Kakamura S., ITano M., Aoyama H., Shimahara K., Yokoyama S., Hirose M. (2003). Comparative Studies of Perfluorocarbon Alternative Gas Plasmas for Contact Hole Etch. Jpn. J. Appl. Phys..

[13] Kim J., Park J., Kim C. (2020). SiO_2_ etching in inductively coupled plasmas using heptafluoroisopropyl methyl ether and 1,1,2,2-tetrafluoroethyl 2,2,2-trifluoroethyl ether. Appl. Surf. Sci..

[14] Kim J., Park J., Kim C. (2019). Angular dependence of SiO_2_ etching in plasmas containing heptafluoropropyl methyl ether. Thin Solid Films.

[15] Betowski D., Bevington C., Allison T. (2016). Estimation of Radiative Efficiency of Chemicals with Potentially Significant Global Warming Potential. Environ. Sci. Technol..

[16] Siepielski A., Morrissey M., Bruno M., Carlson S., Caruso C., Clegg S., Coulson T., DiBattista J., Gotanda K., Francis C. (2017). Precipitation drives global variation in natural selection. Science.

[17] Schipper E. (2006). Conceptual history of adaptation in the UNFCCC process. Reciel.

[18] Sung D., Tak H., Kim D., Yeom G. (2020). A comparative study of C_x_(X = 4,5,7)F_8_ plasmas for dry etch processing. Mater. Express.

[19] Labelle C., Gleason K. (2001). Pulsed Plasma Deposition from 1,1,2,2-Tetrafluoroethane by Electron Cyclotron Resonance and Conventional Plasma Enhanced Chemical Vapor Deposition. J. Appl. Polym. Sci..

[20] Kuboi N., Tatsumi T., Kobayashi S., Komachi J., Fukasawa M., Kinoshita T., Ansai H. (2011). Numerical Simulation Method for Plasma-Induced Damage Profile in SiO_2_ Etching. Jpn. J. Appl. Phys..

[21] Metzler D., Li C., Engelmann S., Bruce R., Joseph E., Oehrlein G. (2017). Characterizing fluorocarbon assisted atomic layer etching of Si using cyclic Ar/C_4_F_8_ and Ar/CHF_3_ plasma. J. Chem. Phys..

